# Graphic analysis of population structure on genome-wide rheumatoid arthritis data

**DOI:** 10.1186/1753-6561-3-s7-s110

**Published:** 2009-12-15

**Authors:** Jun Zhang, Chunhua Weng, Partha Niyogi

**Affiliations:** 1 Department of Radiology, The University of Chicago, 5841 South Maryland Avenue, Chicago, Illinois 60637 USA; 2 Department of Biomedical Informatics, Columbia University, 622 West 168 Street, New York, New York 10032 USA; 3 Departments of Statistics and Computer Science, The University of Chicago, 1100 East 58th Street, Chicago, Illinois 60637 USA

## Abstract

Principal-component analysis (PCA) has been used for decades to summarize the human genetic variation across geographic regions and to infer population migration history. Reduction of spurious associations due to population structure is crucial for the success of disease association studies. Recently, PCA has also become a popular method for detecting population structure and correction of population stratification in disease association studies. Inspired by manifold learning, we propose a novel method based on spectral graph theory. Regarding each study subject as a node with suitably defined weights for its edges to close neighbors, one can form a weighted graph. We suggest using the spectrum of the associated graph Laplacian operator, namely, Laplacian eigenfunctions, to infer population structures instead of principal components (PCs). For the whole genome-wide association data for the North American Rheumatoid Arthritis Consortium (NARAC) provided by Genetic Workshop Analysis 16, Laplacian eigenfunctions revealed more meaningful structures of the underlying population than PCA. The proposed method has connection to PCA, and it naturally includes PCA as a special case. Our simple method is computationally fast and is suitable for disease studies at the genome-wide scale.

## Introduction

It is well known that unidentified population structure can cause spurious associations in genome-wide association studies [1,2]. Such associations typically occur when the disease frequency varies across subpopulations, thereby resulting in the oversampling of affected individuals from particular subpopulations. It is therefore critical to correctly infer population structure from genotypic data when performing genome-wide association studies. Though this topic has been extensively studied, the prevailing methods such as genomic control and structured association still have limitations [3]. Recently, principal-component analysis (PCA) has been employed to summarize genetic background variation [4,5]. Price et al. [3] suggested the inclusion of a few top PCs as covariates in a regression setting to correct for structure. However, there is concern about the interpretation of PCs. Recently, for instance, Novembre and Stephens [6] showed that patterns (such as gradients and waves) appearing in the PC analysis of continuous genetic data sometimes resemble sinusoidal mathematical artifacts. These generally arise when PCs are applied to spatially correlated data. Nevertheless, PCA can provide evidence of major demographic migration events and is still widely used in many contexts for genetic data analysis.

Here we propose a novel approach for detecting population structure inspired by graph theory. Unlike PCA, which uses all pairs of individuals, this method uses the idea of shrinkage and considers only close neighbors as measured by pairwise correlation. Therefore, it is robust to outliers and the results obtained can reveal the local dependence structures of population samples. We demonstrate our method, LAPSTRUCT, on the North American Rheumatoid Arthritis Consortium (NARAC) data provided by Genetic Analysis Workshop 16. Rheumatoid arthritis (RA) is a complex and chronic inflammatory joint disease with both genetic components and environmental factors. It has been observed that *PTPN22 *and *TRAF1-C5 *genes are associated with RA [7].

## Methods

The NARAC study sample includes 868 cases ascertained at RA clinics and 1194 controls from the New York cancer study. The individuals from NARAC were genotyped with the Illumina 550 k single-nucleotide polymorphism (SNP) array in the whole genome, with total 545,080 SNPs. 507,246 SNPs passed quality control after removing SNPs with a departure from Hardy-Weinberg equilibrium (using *χ*^2 ^statistic) in controls significant at the 10^-5 ^level, SNPs with genotype call rates <90%, and SNPs with a minor allele frequency <0.01. Each individual's affection status (unaffected as 0, affected as 1) was regarded as the phenotype. All 2026 individuals in the NARAC data were included in this analysis.

First, let *g *denote the matrix of genotype (0, 0.5, 1) of individual *j *at SNP. We standardize each SNP *i *by subtracting the row mean , and then divide each entry by , where *p*_*i *_is an estimate of the allele frequency at SNP *i *given by ; all missing entries are excluded from the computation. Let *g *still denote the standardized genotype matrix, then . Then, for each pair of individuals *j *and *k*, we define the distance ||*v*_*j *_- *v*_*k*_|| = 1 - *C*_*jk*_. Regard each individual *j *as a vertex *V*_*j *_in a weighted graph G = (V, E), where *j *= 1 to N. Set the weight between individuals *j *and *k *to be a Gaussian kernel  for *j *≠ *k *and ||*v *- *v*_*k*_|| <*ε*, *W*_*jk *_= 0 for *j *≠ *k *and ||*v *- *v*_*k*_|| > *ε *and *W*_*jj *_= 1.0 for all *j*. Here, *ε *is a positive real number that measures the size of each subject's neighborhood in terms of correlations; that is, all individuals within distance *ε *are regarded as one's close neighbors.

Cases and controls are regarded as vertices of a weighted graph and each vertex is connected to its close neighbors through edges according to their pairwise distances. This reflects the fact that distances between vertices that are far apart are relatively less important, and therefore need not be preserved if the sample size of the dataset is reasonably large. The eigenfunctions of the associated graph Laplacian operator on the graph are generalized geometric harmonic functions, which contain geometric structure information of the population dependence graph. The eigenvectors of the graph Laplacian are the first-order linear approximations of Laplacian eigenfunctions. Therefore, they are much more meaningful than the usual PCs as they relate to the intrinsic structure of the data.

Let *D *be a diagonal matrix of size *N × N *with entries , which is a natural measure on the vertices. The Laplacian matrix on graph *G *is defined as *L *= *W*-*D*. Note that *L *is a symmetric and positive semidefinite matrix, and we restrict to the normalized version *D*^-1^*L*, which is not symmetric anymore. The eigenfunctions of the normalized equation *Le *= *λe *are denoted by *e*_*j *_= (*e*_*j*1_, ..., *e*_*eN*_)^*T *^for each *j*, ranked according to the increasing of their corresponding eigenvalues, i.e., *λ*_0 _≤ *λ*_1 _≤ *λ*_2 _≤ ⋯. It is easy to see that 0 is always an eigenvalue with constant eigenvector consisting of all 1 values. These eigenfunctions generalize the low frequency Fourier harmonics on a manifold approximated by the graph *G*. The Laplacian eigenmap with first *n *(usually small, 2 or 3) eigenvectors is defined as *f*: *k *→ (*e*_1*k*_, *e*_2*k*_, ⋯, *e*_*nk*_) for individual *k *to achieve dimension reduction. Note the situation here is different from PCA, where one takes the PCs corresponding to the largest eigenvalues that account for the largest amount of variation in the data.

The Laplacian eigenmap has the important locality preserving property, that is, the distance between a pair of subjects in the Laplacian eigenmap reflects their degree of correlation. The more they are correlated, the closer together they are mapped. Immediately, Laplacian eigenmap leads to cluster-like structures for subjects who either come from the same discrete subpopulation or share more common ancestry in an admixed population. Therefore, we suggest using Laplacian eigenvectors instead of PCs to study population structure. Next we follow Price et al. [3] to regress genotypes and phenotypes on the top ten Laplacian eigenvectors for each individual and compute the adjusted *χ*^2 ^statistic of the residuals.

## Results

**Figure 1:**
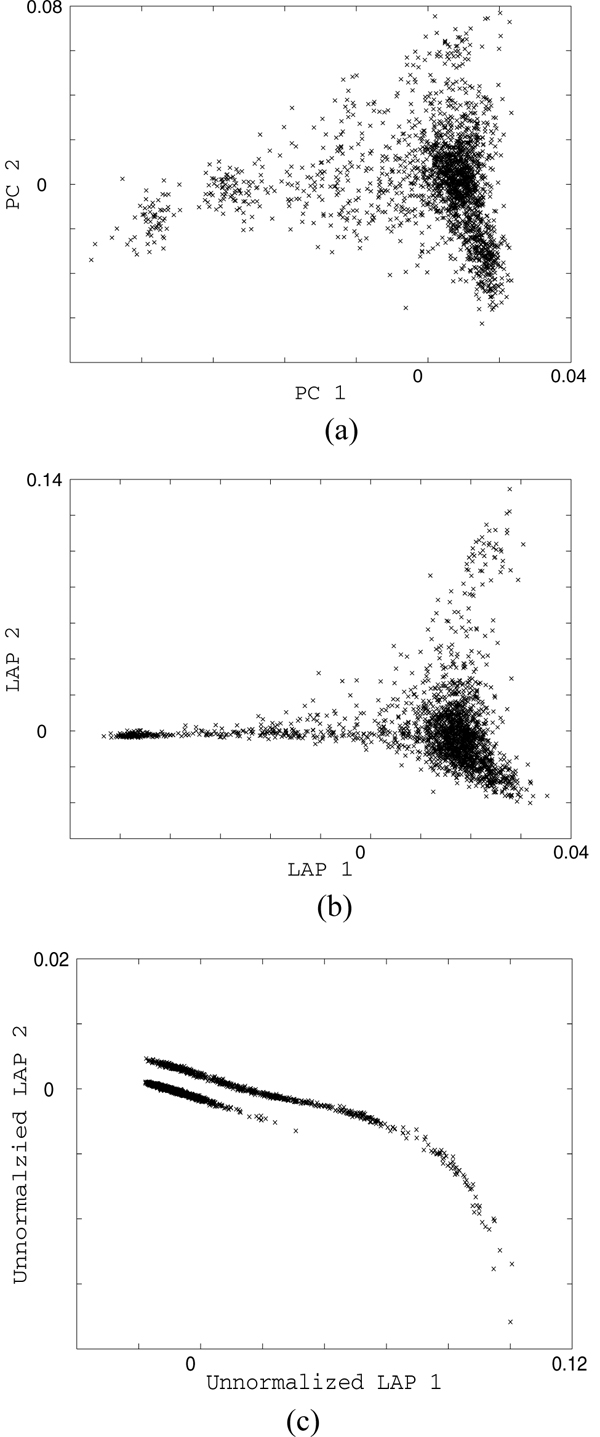
**Population structures**. Detected by PCA: a, Laplacian; b, its unnormalized version; c, both with *ε *= 1.0.

The PC map (Figure 1a) depicts the European population structure similar to the map previously published by Price et al. [3]. The Laplacian eigenmap (Figure 1b) shows the compact trend from center to bottom right and a long tail-like trend to the left. Surprisingly, these two trends are remarkably separated in the unnormalized version of Laplacian eigenmap (Figure 1c). We compared the results for two SNPs that have been reported to be associated with RA (see Table 1). The results are consistent with the prevailing principal-components-based approach, EIGENSTRAT.

**Table 1:** Association testing results for genes *PTPN22 *and *TRAF1-C5 *by EIGENSTRAT and LAPSTRUCT

SNP	Chromosome	EIGENSTRAT	LAPSTRUCT
rs2476601	1	26.74 (2.33 × 10^-7^)	33.72 (6.36 × 10^-9^)
rs3761847	9	27.57 (1.52 × 10^-7^)	25.39 (4.68 × 10^-7^)

## Discussion

By setting a constant weight for each pair of individuals and sufficiently large *ε *to include all individuals into everyone's neighborhood, the proposed approach naturally includes PCA as a special case. This fact follows from the observation below. If all weights *W*_*ij *_are equal, say, , where *N *is the total number of individuals, then  and , where *e *= (1, ..., 1)^*T*^. Let *g *= (*g*_1_, ..., *g*_*N*_)^*T *^denote the genotype data of all individuals, where each *g*_*i *_stands for the genotype vector for the *i*^th ^individual and let *μ *denote the sample mean vector of genotypes. Then one has . Because  is the sample covariance matrix of the individuals, the Laplacian eigenfunctions equal the PCs.

In general, for sufficiently large *ε*, the top Laplacian eigenfunctions describe global variations instead of local dependence structures, and they numerically approximate to the top PCs. As *ε *decreases, the Laplacian eigenmap describes the local dependence structures at different scales. When *ε *becomes so small that each subject's neighborhood shrinks to itself, Laplacian eigenmap cannot detect any structure. In practice, the successful use of the proposed algorithm requires a method to choose effective *ε *to make the graph connected and maintain valid type 1 error for association studies. Similar to the PCA approach for association testing, a method to choose the eigenvector dimension is also required for optimal performance.

We have introduced a novel method for population structure detection that preserves local dependence structures. The Laplacian eigenmap naturally leads to population clusters according to the degree of pairwise correlation among individuals. In our example for testing for association between RA and SNPs, the Laplacian eigenmap method resulted in less noise than the PCA method and detected the same associations between SNPs and RA as the PCA method.

## List of abbreviations used

NARAC: North American Rheumatoid Arthritis Consortium; PC: Principalcomponent; PCA: Principal component analysis; RA: Rheumatoidarthritis; SNP: Single-nucleotide polymorphism.

## Competing interests

The authors declare that they have no competing interests.

## Authors' contributions

JZ and PN designed the algorithm. JZ analyzed the data. JZ, CW, and PN wrote the manuscript.

## References

[B1] Marchini J Cardon LR Phillips NS Donnelly P The effects of human population structure on large genetic association studies Nat Genet 2004 36 512 517 10.1038/ng1337 15052271

[B2] Freedman ML Reich D Penney KL McDonald GJ Mignault AA Patterson N Gabriel SB Topol EJ Smoller JW Pato CN Pato MT Petryshen TL Kolonel LN Lander ES Sklar P Henderson B Hirschhorn JN Altshuler D Assessing the impact of population stratification on genetic association studies Nat Genet 2004 36 388 393 10.1038/ng1333 15052270

[B3] Price AL Patterson N Plenge RM Weinblatt ME Shadick NA Reich D Principal components analysis corrects for stratification in genome-wide association studies Nat Genet 2006 38 904 909 10.1038/ng1847 16862161

[B4] Zhu X Zhang S Zhao H Cooper RS Association mapping, using a mixture model for complex traits Genet Epidemiol 2002 23 181 196 10.1002/gepi.210 12214310

[B5] Chen H Zhu X Zhao H Zhang S Qualitative semi-parametric test for genetic associations in case-control designs under structured populations Ann Hum Genet 2003 67 250 264 10.1046/j.1469-1809.2003.00036.x 12914577

[B6] Novembre J Stephens M Interpreting principal component analyses of spatial population genetic variation Nat Genet 2008 40 646 649 10.1038/ng.139 18425127 PMC3989108

[B7] Plenge RM Seielstad M Padyukov L Lee AT Remmers EF Ding B Liew A Khalili H Chandrasekaran A Davies LR Li W Tan AK Bonnard C Ong RT Thalamuthu A Pettersson S Liu C Tian C Chen WV Carulli JP Beckman EM Altshuler D Alfredsson L Criswell LA Amos CI Seldin MF Kastner DL Klareskog L Gregersen PK TRACF1-C5 as a risk locus for rheumatoid arthritis--a genome-wide study N Engl J Med 2007 357 1199 209 17804836 10.1056/NEJMoa073491 PMC2636867

